# When do physicians perceive the success of a new care model differently?

**DOI:** 10.1186/s12913-021-07061-4

**Published:** 2021-10-06

**Authors:** Simone Richter, Ibrahim Demirer, Maya Nocon, Holger Pfaff, Ute Karbach

**Affiliations:** 1 University of Cologne, Faculty of Medicine and University Hospital Cologne, Faculty of Human Sciences, Institute for Medical Sociology, Health Services Research, and Rehabilitation Science, Eupener Strasse 129, 50933 Cologne, Germany; 2 Department of Rehabilitation Sociology, Faculty of Rehabilitation Sciences, Technical University Dortmund, Dortmund, Germany

**Keywords:** health services research, qualitative approach, relative advantage, outpatient care, diffusion of Innovations, theory of planned behaviour, operant conditioning, care management, case management

## Abstract

**Background:**

The health care innovation “MamBo – people with multimorbidity in outpatient care: patient-focused and needs-oriented healthcare management” aims to improve the efficiency and quality of care for multimorbid patients by delegating tasks (e.g. taking over house calls or coordinating specialist appointments) to a monitoring and coordination assistant (MoniKa). Participating physicians are very important for the success of the health care innovation due to their direct involvement as practitioners and their task of enrolling patients. The aim of this part of the evaluation study is therefore to identify the physicians’ personal values, which influence the individual perception of the project’s advantages and thus possibly the acceptance and sustainable implementation of new care structures.

**Methods:**

Two Focus groups (*n* = 4; *n* = 6) and three individual interviews with general practitioners and specialists who decided to implement the health care innovation within the first year were conducted. The semi-structured guidelines were developed by the research team. The interviews were analysed according to the content analysis by Mayring. We used the learning model of operant conditioning to place our study results in a theoretical context.

**Result:**

Two central personal values of the participants, which determine the desired advantages of the health care innovation were identified: More patient-oriented and more economic-oriented values. Participants with more patient-oriented values quickly perceived advantages, which seems to be beneficial for the acceptance of the new care structures. Economic-oriented participants tended to be more critical. The benefits of the health care innovation, which was expressed, for example, in an improvement of the practice routine, has not yet been perceived by this group, or only to a limited extent.

**Conclusions:**

The results suggest that the respective values of the participants define the individual perceived advantages and thus, the assessment of the success of the health care innovation in general. These findings could be used in the implementation process by increasing the motivation of the project participants through typified supervision.

**Trial registration:**

The study has been registered in the German Clinical Trials Register (DRKS00014047).

**Supplementary Information:**

The online version contains supplementary material available at 10.1186/s12913-021-07061-4.

## Background

Demographic change will lead to ageing of society and, in this context, to an increasing proportion of people suffering from several chronic diseases [1]. About one in three adults suffers from more than one chronic disease, which is even higher in developed countries. Cardiovascular disease, cancer, respiratory disease and diabetes are the major non-communal diseases, accounting for three out of five deaths worldwide [2].

The development of an ageing society and the accompanying increasing burden of non-communicable diseases, chronic conditions and multimorbidity will lead to an increasing demand for health services. Health care spending rises sharply with each additional chronic condition, as for example more specialists, emergency rooms and hospitals have to be visited. The burden on patients is also enormous and associated with a deterioration in quality of life. Thus, out-of-pocket payings increase, medication becomes more complex, and patients may become unable to work [2].

The health care of chronically ill and multimorbid patient’s is complex and requires interprofessional cooperation between general practitioners (GPs), specialists, the inpatient sector and nursing care [3, 4]. Patients with serious illnesses or chronic conditions and thus with complex care needs often report a lack of integrated and well-coordinated care - by which transparent and effective communication between the different care providers involved and clear responsibilities is meant. A lack for example is shown by a survey of patients with complex care needs in eleven countries, besides other including Canada, Germany, France, the Netherlands, the United Kingdom and the United States [5]. The patients’ reports show that the flow of information between the different settings, e.g. during transitions from hospital to outpatient care and between general practitioners and specialists, is often not sufficiently guaranteed. The review of medication and receiving test results in a timely manner were also mentioned as common problems in the countries [5].

When different professions and disciplines are involved, it is often unclear who is responsible for patient coordination. This is where the GPs play an important role [3, 5, 6]. As one way of assigning this responsibility more clearly, many countries have a gatekeeper system. In this system the patients have to visit a GP first, before seeing a specialist or getting acess to a hospital. Thus, in the role of gatekeeper, GPs can on the one hand contribute to cost control by reducing “unnecessary” interventions, and on the other hand coordinate secondary care more efficiently, as GPs usually have better information [7]. In Germany, there is no overall legal regulation for it. GP-centred care currently takes place only in rudiments (§ 73b SGB V) [8]. Nevertheless, GPs in Germany play an important role in the coordination especially of older multimorbid people. However, there is a lack of a holistic and cross-indication perspective as well as a lack of resources. Many non-medical tasks associated with the care of multimorbid patients are not paid to the physicians and cost a lot of care time [9]. McKinglay et al. pointed out that, although general practitioners are believed to be best placed to coordinate the care, the situation is further complicated when several professionals and, in addition, authorities are involved [6]. Besides the clinical difficulties faced by general practitioners in the care of multimorbid patients, the load from non-medical tasks is increasing, and delegation of tasks is therefore playing a greater role [10]. To meet these challenges, scientists and health professionals are working on the development, implementation and evaluation of care managment innovations. Many international researches focuses on the Chronic Care Model (CCM), an evidence-based approach to improve the care structures for chronically ill patients [11] or on nursing-led interventions in the home setting, where nurses take over deligable medical tasks [12]. Just as in a systematic review, that also included four German studies, on care management interventions in dementia care, the included studies often focus on a symptom or the disease itself [13].

 In line with this, but with a more intersectoral and cross-disease approach, the care model “MamBo- People with Multimorbidity in Outpatient Care: Patient-Focused and Needs-Oriented Healthcare Management” has been developed. MamBo had been implemented in July 2017, in the context of a physician’s network in the region of Leverkusen, a small metropolis in North Rhine-Westphalia (Germany). The core element of MamBo is a cross-sectoral coordinated case management of multimorbid patients – conduceted by monitoring and coordination assistants (MoniKas) – set up in a collaborating Regional Health Network (RGL). Participating GPs can delegate housecalls to the MoniKas, which are trained nurses, to support the care of multimorbid patients. At the patients’ home, the MoniKas first assess the needs of the patients, including medical, nursing and social-legal needs. Then the MoniKas take over the coordination with numerous actors in the health and social care sector while being in regular contact with the treating physicians. With this comprehensive information GPs can provide better patient-centred care, make more purposefull prescriptions and reduce their own workload. The GPs can decide who gets a MoniKa according to their own estimation - these are often elderly, lonely, multimorbid people with limited independence. Only physicians who are part of the physicians’ network can participate in MamBo. Therefore, specialists could also have used a MoniKa and enrolled patients. However, during the implementation process, it became clear that specialist did not play a role in the use of the intervention, but rather a role as ambassadors in disseminating the innovation. Until October 2020 41 out of about 100 potential physicians participate in the project. The implementation of the health care innovation “MamBo” is accompanied by an evaluation study with a project duration of three years and nine month (July’17 – March’21). Further information on the evaluation study of MamBo can be found in our study protocol [13].

As participating GPs are in charge of delegating house calls, caring for and enrolling patients, their acceptance of the new structures is essential for the successful and sustainable implementation of healthcare innovation. Experiences show that the implementation of innovations, like the establishment of case management, often takes a long time and remains a challenge, especially concerning achieving adoption in the primary care setting. In this respect, it is crucial to understand which factors influence the sustainablilty of an implementation of complex health care innovation [14–16].

According to Roger’s Diffusion of Innovations Theory [17], the relative advantage desired by potential users is a characteristic of an innovation that strongly influences the individuals’ decision to implement it. It is the degree to which an innovation is perceived as being better than the idea it replaces. In the theory of planned behaviour, the behaviour is determined by behavioural beliefs, and the perception of positive or negative outcomes through the behaviour form the behavioural beliefs [18]. Accordingly, well-known theories referred to implementation research show that the relative advantage is an essential factor for the implementation of an innovation. Innovations that have a decisive advantage over the conventional standard are more easily adopted and implemented [17, 19, 20].

Not only theoretical but also current research evidence indicates that the relative advantage is an essential factor for the adoption of an innovation [16, 20–22]. In a quantitative analysis of the association of certain attributes on physicians’ intention and actual use of an intervention, Scott and colleagues [21] found two of its qualities to be more influential than the others, namely relative advantage and observability. They also found out, that solo physicians had higher environmental and idividual barries for the intention to use an intervention, compared to group practices. Thus, work context can helpshape the innovation adoption process. The benefit of e.g. the implementation of a new care model conceptualize differently between the potential adopters for instance in terms of economy, social reputation or user-friendliness [20, 21, 23]. Denis and colleagues pointed out that potential adopters do not act in an entirely rational manner, but according to their interests or values and power dependencies. Their observations suggest that change can be more successfully driven if it can be based on values that legitimise the chosen position – and these values for example can and cannot be based on scientific evidence. While in one study listed, a leading clinician referred to research and literature as a source of evidence, for others, compliance with standards of care was seen as the most important value [23]. Also Greenhalgh et al. identified in their systematic review cognitive and social psychology factors such as the individual’s motivation, values and learning styles, which influence the implementation of innovations [20].

Furthermore, after implementing a new innovation, participants must decide whether to continue or discontinue the adoption [17]. In our case, physicians may also choose to take a more passive role and not, for example, continue with all aspects of MamBo such as patients’ enrolment. By personal values we understand factors (e.g. scientific knowledge, money, social prestige) that are important to participants, motivate them and guide their decisions. Personal values seem to be a key element in the decision-making process, namely the basis of what individuals want to see as the benefits of an innovation. Based on the fact that relative advantages play a driven role in the implementation of innovations, this study aims to identify the personal values of physicians participating in MamBo. We further address the question of how this may affect a sustainable implementation of the new care model in our study population.

## Methods

### Study design

Since little is known, we used an explorative research design to identify personal values and expected advantages. In the context of the formative evaluation of the evaluation study MamBo, interviews with MamBo-physicians were conducted. The interviews were performed in the form of focus groups, which were supplemented by face-to-face interviews. This study shows the results of the focus groups and qualitative interviews based on qualitative content analysis with both a deductive and inductive approach [24, 25]. The Diffusion of Innovation Theory by Rogers was stated to underpin the development and analysis of the interviews [17]. We have further used the learning model of operant conditioning to place our study results in a theoretical context and discuss the link between personal values, a rapid perception of advantages and a successful implementation process [26, 27].

### Sampling

The recruitment of physicians was based on the Theory Rogers “Diffusion of Innovation”, i.e. physicians that were active from the beginning (“early adopters”, within the first year) where interviewed separately from physicians that became active later (“late adopters”) [17]. As this study is based on early adopters, all participating doctors who took part within the first year after the start of the MamBo project (*n* = 25 of 41) formed the study population. Due to the small study population, other characteristics, such as gender, could not be taken into account when compiling our sample [28]. Physicians who had participated once couldn’t participate in the formative evaluation again. The board of the care management provided access to the 25 physicians, by handing out invitation letters and information and reminding the physicians to respond. The invitations and information letters explained the objectives, ethical aspects and procedure of the study, and a letter of consent were sent via fax and e-mail. Also, an expense allowance (120 €) was offered for participation [28]. One focus group was conducted in June 2018 with six GPs. A second focus group took part in January 2019 with three GPs and one specialist (neurologist). Since three doctors expressed their interest in being interviewed, but no common date for a focus group for all suitable could be found, the second wave was supplemented by individual face-to-face interviews with two GPs and one specialist (pneumologist). In all, the results are based on data of 13 physicians (responserate: 53 %) who have been physicians in residential practice for around 20 years in average. Further details and characteristics can be found in Table 1. All participating doctors have used the service of the case management at the time of being interviewed, i.e.they had called in a MoniKa at least once. While three physicians were less active based on their statements that they did not use the MoniKa structures frequently and had enrolled very few patients at the time of the interview, four physicians in particular seemed to have used the structures quite quickly and with high commitment. From the statements of the others, no special, neither inactive nor particularly active behaviour could be assessed.

**Table 1:** characteristics of the sample and information on the conducted focus groups / interviews

	n	Start of participation in MamBo	Date of the FG	Duration FG
Focus group 1	*n* = 6 **sex**: f:*n* = 2; m:*n* = 3 **GP**: *n* = 6	sep’17: *n* = 3 oct’17: *n* = 1 nov’17: *n* = 1 feb’18: *n* = 1	June 2018	86 min
focus group 2	*n* = 4 **sex**: f:2; m:2 **GP**: *n* = 3 **Specialist**: *n* = 1	oct’17: *n* = 1 nov’17: *n* = 2 feb’18: *n* = 1	Janurary 2019	72 min
Individual Interviews	*n* = 3 **sex**: m:3 **GP**: *n* = 2 **Specialist**: *n* = 1	sep’17: *n* = 1 oct’17: *n* = 1 nov’17: *n* = 1	Janurary 2019	30–60 min

### Data collection

The interviews followed a semi-structured guideline [24, 28], which was developed by two well-trained researches (SR, UK). The interview guide includes a total of five main topics, of which the following are related to the aim of the study:

- Intention to participate in the healthcare innovation.
- Implementation of the healthcare innovation and its evaluation.
- Perceived changes in daily work routine: advantage or disadvantage of the innovation.

For each main topic, open questions were designed to generate narratives from the participants. The interview guide was flexibly adapted to the course of the conversation. Adaptations of the guide, according to the background and type of physician, were made prior to the interviews [24, 28]. The guidelines that were used for the focus groups are attached as additional file (see Additional file 1). For the individual interviews, the guide from focus group 2 was used and slightly adapted.

To take field notes, two research assistants took part in the first focus group in addition to the moderating researcher and one assistant in the second focus group. The focus groups were conducted in the premises of a regional physicians’ network. The face-to-face interviews were held in the physicians’ practices. All interviews and focus groups were audio- recorded and ruled led transcribed and pseudonymised [29]. Right after a focus group or interview, a memo was written by the interviewee to obtain supplementary and contextual information for the analysis [28].

### Data analysis

To analyse the interviews, qualitative content analysis was used [25]. We have defined to consider mainly manifest content in the analysis, but also general agreement and disagreement among participants on individual statements [30]. We also tried to examine underlying meanings to identify personal values and interrelations between statements. Thus, we filtered the value orientations based on stated interests and reasons for participating in the projects, as well as on values and norms stated in relation to their work attitudes. At the end we classified our interview partners according to identified value orientations and compared their statements regarding their perceived advantages and their assessed success of the project. To get completely familiar with the data two researches (SR, UK and a research assistant in various combinations) read the transcripts intensely before conducting the initial data analysis. The coding of the material was conducted in two steps. First, main categories and subcategories were defined deductive, based on the guideline topics and Rogers’s theory “Diffusion of Innovations” (e.g. main categorie: Intention to participate; supcategorie: MamBo as a solution for patients related challenges). The resulting codebook, including definitions, coding-rules and examples out of the material, were developed by the first author and revised by UK. In the second step, this codebook was used to initially code the material by an inductive approach. Inductively developed codes either fit into a deductively defined category or were included as a new category in the codebook. The codebook was repetitively discussed and revised among the researches until consensus was obtained. The final codebook includes a short description for each code which is relevant for the research question. Further interpretation was performed by the first author and reviewed by as well as discussed with UK. The coding was assisted by the use of the computer software MAXQDA (VERBI GmbH, Berlin, Germany). Based on the theoretical assumption that personal values determine relative advantage, we analysed participants’ statements about their intention to participate in order to determine their personal values from these.

## Results

The main categories “Compatibility”, “Intention to participate”, “(not) perceived advantages of the innovation” and “System-related challenges for implementation and transfer” include in total 22 subcategories relevant for the research question. A detailed description of the categories and their definitions is added additionally (see Additional file 2). The personal values and their relation to the perception of advantages as well as the assessed success of the healthcare innovation were analysed by comparing statements that were assigned to the four categories mentioned above and their subcategories.

### Values determining the perception of advantages

All informants acknowledged the work done by the MoniKas, in particular the assumption of non-medical, but social management tasks. All interviewed doctors received positive feedback from patients and their relatives and emphasized their satisfaction with the visits of a MoniKa - for example, in terms of patients feeling better informed and better cared for socially. But also, the challenges of a future transfer of the MamBo-structures to the standard health care system in germany, such as the question of how to cover necessary financial and personal resources, were addressed in all interviews^1^. Other challenges mentioned in the implementation were the bureaucratic burden of enrolling patients, the cooperation with insurance companies and their interest in attracting doctors to other and similar projects, and the limited time for noticeable changes.

Even if these potentials and challenges are recognized by almost all interviewees, the evaluation of these according to the personally perceived advantages is different. Thus, we found that the desired relative advantage, as most relevant for implementing the healthcare innovation, were mainly determined by (a) patient-oriented values or (b) economic-oriented values - taking into account that people with stronger economic-oriented values can also be driven by patient-oriented values or vice versa. However, certain values have a greater priority than others, which then determines the mainly desired advantage. Table 2 provides a sample case for perceived challenges, perceived benefits for patients and relative benefits for the doctors themselves, per group. We also identified further values and factors influencing the intention to participate (e.g. social norms, former project experience), which, however, do not seem to determine the desired advantage, or not in a substantial way [23]. We now present the characteristics of participants with patient-oriented and economic-oriented values.

**Table 2:** Exemplary comparison of the perceived challenges, noticed MoniKa interaction and the relative advantage per value-orientation group

	Challenges	Noticed MoniKa-interaction	Relative advantage
Patient-oriented value - Case example BB	- unpleasant to convince patients to participate and time required - Limited staff in own practice. - multiple and parallel projects and technologies - bureaucracy - complexity of enrolment documentation	- helpful in social care/ social rights (e.g. applications for power of attorney; care grading) - positive feedback by patients who has been visited by a MoniKa	tangible positive changes based on the following relative advantages: - improvements in patient care - non medical tasks - care of relatives - medical care at home - strengthening practice towards patients
**Economic oriented value – ** **Case example Exp2**	- limited time for noticeable changes - multiple and parallel projects and technologies - external control - bureaucracy - complexity of enrolment documentation - convincing patients to participate - limited personal ressources in case of transfer to standard care	- helpful in social care (application for severe disability; provision of medical aids, care grading, applications) - positive feedback by patients who has been visited by a MoniKa	positive changes based on the following relative advantages not yet noticeable: - relief of workload - monetary effect

#### (A) patient-oriented values

Doctors interviewed, who had strong patient-oriented values (*n* = 5), emphasized the importance of holistic patient care (medical, emotional, social) and were very concerned about the care of elderly people living alone at home and experiencing poor social support.

*“And indeed, there are many ways. It doesn’t have to be MamBo. The main thing is that the patients are well cared for.” (Exp. 03)*.

They also felt that the new care model could be a solution to the challenges associated with the medical care of multimorbidity, such as reduced mobility, communication problems and low compliance of multimorbid patients.

*“[…] cognitive impairment of the patients, which lead to the fact that they are not in compliance with instructions, as we wish as physicians. In addition, mobility is becoming increasingly limited, which makes communication with patients who otherwise regularly come to the practice more difficult. The decreasing support from the family, which does not exist in small families. The wife is there because children often move far away, if there are children at all.“ (FG 1. BB)*.

#### (B) economic-oriented values

We identified that the desired advantage of participants with stronger economic-oriented values (*n* = 2) was mainly based on monetary interests and interests to improve processes within their practice. The focus was on the cost-effectiveness of the new model, which was assessed by comparing the resources used to implement it with, for example, the reduction of workload or hospital stays of patients. Thus economic-oriented values could manifest not only in the form of personal cost-benefits but also in the interest of reducing social costs.

*“I might have to say again that all the non-medical task we do here, they don’t get paid. Yes, we do it all for free. And who will do that in the future? I don’t see that. And then what will the care landscape look like, so it is really urgently necessary to have something like this.” (FG1, GG)*.

*“The second is, I believe, that it is very important that money flows into this area. If there is no economy, they can forget everything. That is daydreaming. We have that in masses behind us.” (FG1, DD)*.

Table 3 includes identified factors which determine patient-oriented and economic-oriented values.

**Table 3:** Conceptualisation of patient- and economic- oriented values

patient-oriented values	economic-oriented values
• social management • drug management • patient information • patient satisfaction • patients’ security • continuous care	• cost reduction • practice procedure • social costs

### Perceived advantages and evaluation of the projects’ success

Participants with more patient-oriented values experienced the benefit for the patient also as an immediate personal advantage.

*“Well, that’s what I meant in the first place. Seldom something like that is so well accepted. The patients call and are so happy that they are in the project. Such statements are made spontaneously. And I didn’t hear anybody say, well, listen, that’s nothing or something like that. Never. Not once. Well, in that respect, I can only say positive things, yes.” (Exp 01)*.

*“And with such a positive tailwind, which they bring with them because, as I said, they feel that they are in good hands, things run easier. And safer.” (FG 2, AA)*.

These participants felt a work relief through the delegation of home visits, although most of the tasks undertaken by MoniKa were social-management rather than medical. They communicated a perceived advantage in terms of patient safety. On the one hand, they expressed that they have an advantage from the fact that their patients are safer at home when someone trained has taken a look at the home environment (see also Table 3).

*“I think this is useful for me, too, when I know that patients are at least safer at home. There is no longer the tripping hazard of the carpet, there is perhaps also a nursing classification that is now happening here. There’s someone who looks to see if a severely disabled person’s ID card is necessary or something else.” (FG1, BB)*.

On the other hand, they feel better when they know that their patients are being cared for safely, for example, during their practice holidays. By delegating tasks to MoniKa’s continuous care can also be ensured in that case.

*“That there is a continuity of care when you are on holiday, that the patient does not have to go to the substitute doctor. The patient is overstrained with such a big thing.” (FG1, EE)*.

Furthermore, participants with strong patient-oriented values quickly perceived advantages, shortly after implementation. They were more optimistic about the success of the project and spoke very positively about the new care model as a whole. Also, they reported that the enrolment of patients also became more straightforward when a direct benefit was noticed.

*“Basically, I specifically addressed those where I saw that they would directly benefit from it. And after I noticed that it actually works well, that it is actually a good offer, it was much easier.” (FG2, DD)*.

In contrast, participants with more ***economic-oriented values*** did not see a direct advantage for themselves from the patient benefits, and they experienced (only) little connection to the success of the project in general (see also Table 3).

*“Nope, so, a care level has now been classified in a case or one or the other care aid has been purchased. Well, these are then improvements for the patients in the care level. They could also pay someone or…that’s something concrete, yeah. And beyond that I wouldn’t know right now if something has changed.” (Exp. 2)*.

*“So, for me the use of MamBo is very difficult to evaluate, because of course the problems remain in my memory, where something doesn’t work. And when I have hired MamBo like MoniKa, I get a feedback and I think it’s very positive, but I don’t notice a direct advantage for me“. (FG1, AA)*

For example, one participant with strong economic interests did not see any relief in his work, although he recognized and positively mentioned the work of the MoniKas who conducted home visits. He explained this by the fact that so far too few patients of his practice are involved in MamBo and have received a MoniKa.

*“Well, […] nothing worth mentioning has changed. Because we have two and a half thousand patients a quarter and from this 40 are, or, I don’t know, maybe a little more, are in the MamBo project. Well, that is an amount that is not really worth mentioning. And the patients, for some of them the one or other advantage resulted from the visit of MoniKa, that was quite helpful.” (Exp. 2)*.

**Fig. 1:**
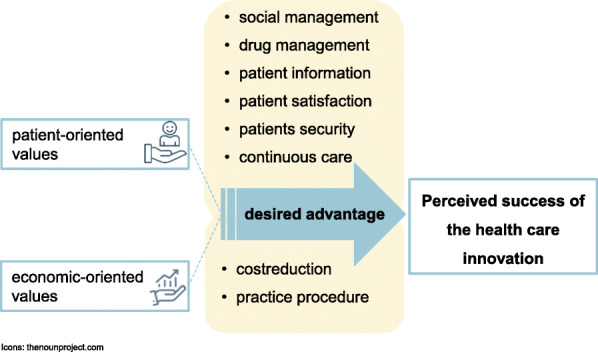
Theoretical model of the link between personal value orientations, desired advantages, and perceived project success

So far, no changes in daily practice or at the societal level have been noticed in the outcomes relevant to them. They were less convinced of the new care model and expressed scepticism, especially with regard to proof its cost-effectiveness within the limited study period. Figure 1 models the link between the desired advantage, depending on the personal value orientation, and the perceived project success. Furthermore, negative consequences, such as the current expenditure of resources were more present in the interview when participants represented more substantial economic interests.

*“Well, for that, MamBo would have to prove that it’s somehow cost effective. I think it’s going to be very difficult. It’s going to be hard, just because of the amount of staff involved. I can hardly imagine that it will be successful in the end. Or it is still way too early for that or there are still too few people included. Well, you really should be able to prevent a stay in hospital or perhaps improve the medication etc. somehow, so that people really get a better care and have to go to hospital less often. And I’m sceptical about whether that will succeed. We’re all sceptical about that, I suppose.“ (Exp. 2)*.

*“Nah, I don’t see any relief. So what perhaps relieves me is that MoniKa now makes house calls and makes useful proposals, which I think is good. But I have more documentation and communication work to do.” (Exp. 2)*.

## Discussion

### Findings in the context of a theoretical approach and existing research

 This study aimed to identify physicians’ values, which influence the individual perception of the success of the health care innovation and thus possibly the acceptance and dissemination of new care structures. Following what we found in the analysis, differences in a perceived success of the health care innovation could be explained by different personal values which determine the desired advantage by implementing the new care model. Even if positive changes for patients are recognized, this does not necessarily led to an sustainable implementation of the new care structures by participating physicians. Based on our findings and with reference to a theoretical learning approach, we assume that quickly noticeable advantages are promotive for a continuous as well as a sustainable implementation. Accordingly, relative advantages that only become noticeable after a more extended period, such as economic benefits, inhibit the perception of the project’s success and its continuous implementation. The approach of operant conditioning can be used to support our assumption. As long as a positive consequence is expected or occurs, it is more likely that the behaviour will be repeated. However, the shorter the time between the behaviour and its consequence, the stronger the effect on the repetition of the behaviour [26, 27].

**Fig. 2:**
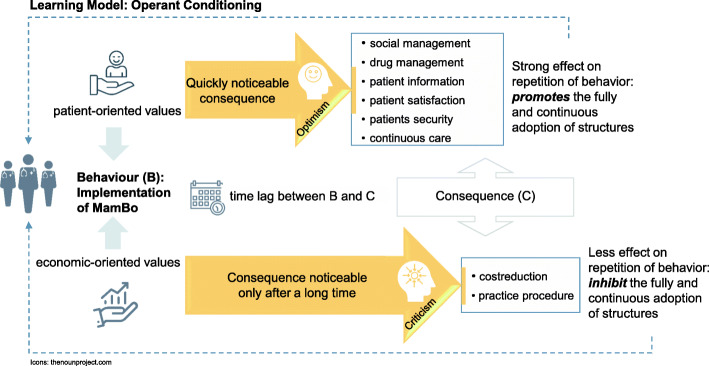
Application of the learning model operant conditioning to the study results

Figure 2 shows the transfer of the approach to our study results. The behaviour “Implementation of MamBo” should be repeated, or in our case, continued. The perceived advantages, conceptualized by the respective outcome relevant for either patient-oriented or economic-oriented participants, are the consequence of the behaviour.

Following this approach, MamBo participants with stronger patient-oriented values would be more likely to continue the adoption of the MamBo structures as the relevant advantages for them are quickly noticeable after implementation. Moreover, optimistic and convinced participation promotes communication and thus, the diffusion of innovation [17]. In contrast, it is less likely that economic-oriented participants will continue the implementation in its complete form. Since so far, no or only little advantages have been perceived, no desired consequences reinforces the behaviour.

Our findings support the observations of Denis and colleagues in their multiple case study, namely that the perceived advantage is based on individual interests or values, such as economic interests, social prestige or to follow best clinical practice [23]. The findings of our study also support the suggestion of Scott and colleagues that the advantage relative to a participant is conceptualized differently by those potential adopters [21]. As Greenhalgh et al. found out, it is more likely that potential adopters will use an innovation, if it meets their needs [20].

### Implications and contributions to the field

As personal values predict the relative advantage of an innovation, it gives an indication of how the individual participants would evaluate the success of the health care innovation. By considering the personal values of potential study participants already during the planning and implementation of new care structures, a significant contribution can be made to the successful implementation of them. Typified support could increase the motivation of the participants to adapt to new structures.

By using a qualitative approach, personal values and intentions for participation as well as the complexity of the innovation were revealed, which would have been difficult to identify with quantitative methods. While studies use the theory of operant learning to improve the adherence of treatments, the application of a learning theory in implementation science seems quite rare - although the adoption of new structures is a complex learning mechanism. The use of a learning theory like operant conditioning helps to understand and explain phenomena and dynamics in the adoption process [26, 27].

Our study is another example of the importance of considering personal values of the target group when assessing new innovationein health care. It contributes further to answering the question of why some less robust interventions are widely used and others with stronger evidence are not. By applying our findings to the operant learning model, our study also contributes to explaining this phenomenon and we extend previous research to include the temporal component. Quickly noticeable benefits, if addressed based on personal values, are more likely to lead to continued adoption of innovations in healthcare. Therefore, it is helpful to look at related areas and transfer theories from psychology to implementation research in healthcare.

### Trustworthiness and limitations

Our study is mainly limited by a small study population, but also by inconsistencies in interview formats and the long period between the two focus groups. This study is based on 2 focus groups and 3 complementary interviews, which wasn’t the origin idea. Initially, purposeful sampling was planned to achieve a variation of the participants in terms of sex and work experience within the group of early adopters [24, 28]. However, since we were unable to convince as many physicians to participate in the project as planned, the pool of potential participants for a focus group was smaller than assumed. Thus, we had to consider any interested physician for participation in our study independent from sex and work experience. Due to a communicated lack of time of the doctors and difficulties to obtaining an answer from the doctors, even with the support of the board of the Care Management, we could not win more participants for different focus groups in either of the two waves. To be able to collect more data, we had to conduct supplemented face-to-face interviews, which makes it difficult to compare the statements as the context is different. We interviewed only four female doctors and two specialists. However, the specialists did not play a major role in the implementation of the innovation, as it became clear during the implementation phase that MamBo’s structures are geared towards primary care. The associated increased risk of selection bias and an incomplete, as well as a small sample in our study, limits the credibility and transferability of the results. It is also possible that the participants in the first wave experienced the implementation process differently from those in the second survey wave. In any case, the participants of both groups were defined as “early adopters”. In addition, the results of our study are limited by the lack of triangulation and linkage with data on the frequency of commissioned MoniKas or the number of enrolled patients by our interviewed physicians.

Based on previous research, we have focused on relative advantage, as this factor is known to be one of the most critical determinants for the implementation and diffusion of an innovation [16, 20–22]. Other determinants of implementation were not considered. Besides the importance of personal values and the time lag between implementation and consequence, numerous other factors influence the successful implementation of an innovation (e.g. complexity, trialability and observability of an innovation, information, support) [31]. The interpreted data are trustworthy in that three researchers read the transcriptions, participated in the discussions about the coding and reviewed the interpretations [30].

## Conclusions

This study contributes to the investigation of the determinants for the successful implementation of new forms of care. Our results suggest that the respective personal values of the participants can predict the individually perceived success of the health care innovation and that a quickly perceived advantage may affect the sustainable implementation of e.g. a new healthcare model. Since this is a theoretical assumption based on the subjective perception of individual participants, further investigations with a more extensive study population must be carried out, including quantitative process data such as the number of enrolled patients or satisfaction in people with different value orientations. Future research is encouraged to use learning models as theoretical constructs for implementation research since implementation is a complex learning process.

## Supplementary information

Additional file 1. Interview-guideline focus group 1 and Interview-guideline focus group 2. The additional file 1 includes the Interview-guidelines used in the first focus group and the interview guideline used in the second focus group, which were used and briefly adapted for individual interviews as well.

Additional file 2. Final codebook. The additional file 2 indcludes a detailed description of the main categories, subcategories and their definitions.

## Data Availability

It is planned to submit the results of the formative and summative evaluation for publication in peer-reviewed journals and to present them at national and international conferences. The dissemination will also be supported by professional public relation activities. The anonymous datasets generated during the current study may be made available from the corresponding author on reasonable request. Protocol modifications will be communicated to relevant parties. such as the publisher of this study protocol or the trial registry. The study manager will oversee the intra-study sharing process. All project members listed in this study protocol will have access to the cleaned anonymous data sets of their respective work packages.

## Abbreviations

CM: Care management
DM: Demand management
GP: General Practitioners
MoniKa: Monitoring and coordinating assistant
MamBo: People with Multimorbidity in Outpatient Care: Patient-Focused and Needs-Oriented Healthcare Management

## Footnotes

1 Health innovations such as MamBo, which are financed by the federal innovation fund, are evaluated for their effectiveness using primary and secondary quantitative data in order to transfer the structures to standard care in Germany if they are suitable and effective.
