# Supplementary material for: When do physicians perceive the success of a new care model differently?
Source: BMC Health Serv Res. 2021 Oct 6;21:1058. doi: 10.1186/s12913-021-07061-4 (PMC8495962; doi:10.1186/s12913-021-07061-4)
Supplement: Supplementary file 1 — Interview-guideline focus group 1 and Interview-guideline focus group 2. The additional file 1 includes the Interview-guidelines used in the first focus group and the interview guideline used in the second focus group, which were used and briefly adapted for individual interviews as well. [file 12913_2021_7061_MOESM1_ESM.docx]

**When do physicians perceive the success of a new care model differently?**

A qualitative study on personal values of physicians and their expected advantages through the implementation of a new care model in a primary care setting

Authors: Simone Richter^1^, Ibrahim Demirer^1^, Maya Nocon^1^, Holger Pfaff^1^, Ute Karbach^2^

1) Institute of Medical Sociology, Health Services Research, and Rehabilitation Science (IMVR), Faculty of Human Sciences and Faculty of Medicine, University of Cologne, Germany

2) TU Dortmund University, Faculty of Rehabilitation Sciences, Germany

**Authors information**

Ibrahim Demirer: [Ibrahim.Demirer@uk-koeln.de](mailto:Ibrahim.Demirer@uk-koeln.de)

Maya Nocon: [Maya.Nocon@uk-koeln.de](mailto:Maya.Nocon@uk-koeln.de)

Holger Pfaff: Holger.Pfaff@uk-koeln.de

Ute Karbach: ute.karbach@tu-dortmund.de

**Corresponding Author**

Simone Richter

Eupener Strasse 129

50933 Cologne

Germany

Tel.+49(0)221/478-97158

Fax +49(0)221/478-1441464

Simone.Richter@uk-koeln.de

## Additional file 1:

## Interview-guideline focus group 1

| **Topic** | **Procedure** | | | | **Notes/ Intention** | |
| --- | --- | --- | --- | --- | --- | --- |
| **Welcoming (5min)** | Thank you for participating in MamBo and having the time to join the focus group. As you all know, MamBo aims to improve the outpatient care of multimorbid patients. Whether, how and why MamBo could succeed or not remains to be clarified. For this reason we have invited you today. | | | | - Entry - Introduction Mambo | |
| **Framework (5min)** | The focus group will take around 90min. At the end there will be a catering. I will record the discussion on tape as announced so that we can evaluate your statements scientifically afterwards and do not have to rely on our memory. At the end I would like to ask you to fill out a short questionnaire about yourself, this is about socio-demographic data and how long you have been participating in Mambo.  All personal data in the interviews will be anonymized, so that no conclusions about your person or your patients will be possible. My two colleagues here will note down the change of speaker during the discussion, which will make it easier for us to transcribe later.  Do you have any further questions? | | | | - Explanation of the frame - handing out short questionnaires - Presentation of the moderator and protocolants - Consent for recording | |
| **topic** | **Main Question** | **Check** | | **Concrete request** | **Notes/ Intention** | |
| **3. Warm-Up**  **(10-15 min)** | Imagine multimorbid patients in your practice. What are the main problems with their care in the outpatient sector? | - challanges in the care of multimorbid patients (medical, emotional, coordination - Reasons for the participation | |  | - Create an emotional entry and framing here. - Collect the main points of criticism here and rewind them in relation to Mambo. | |
| **4. Implementation and evaluation of the MamBo structures (approx. 30 min)** | | | | | | |
| **Expectations** | *On which aspects of the above-mentioned, in your opinion, should the mambo care model be based?* | - expectations - Target group - particular indications - Expenses for enrollment | | - *Which patients would you recommend Mambo to?* - *Which aspects of Mambo are you particularly interested in?* - *Is there a difference between your expectations and reality so far?* - *On which aspects does the Mambo care model actually focus?* - *Why do some aspects come too short?* | | - Intention to participa - Recording the expectations of the model. - Implicit evaluation of the care model by contrasting between - Actual & target state. - Discussing the aspects that have been neglected and the reasons for this. |
| **Cooperation with the MamBo-actors** | *How is the cooperation between you and the other actors of Mambo?* | - Contact regularity - physicians network, insurance - Specialists (network file) - Contact with social services | | - *How do you assess the cooperation with the care and demand management* - *How do you perceive the role of the insurance?*   *(With a view to the roles: Initiator/Impusher/Occupier?)* | | - Role of the insurance (initiator/ influencer/ occupant) - Contact persons and responsibilities CM and DM helpful and useful? |
| **Continuous improvement process (CIP)** | *How did you perceive the impact of management consulting?* | - change managament in the practice - Workshops | | - *Did the offer of your involvement in CIPs appeal to you?* | | - Awareness of the CIP contents - Evaluation of management cosultancy |
| **5. changes in everyday practice - reflection/ results (15 min)** | | | | | | |
| **Change in everyday practice life?** | *What has changed in your daily practice due to the participation in Mambo?* | - Changes positive? - Existing relief? - Additional effort - For example in relation to: enrollment, Monika, drug management, network file, documentation, cooperation RGL | | - *Are you satisfied with the changes?* | | - Especially Monika should be focused and emphasized here... |
|  | *What experiences have you made with the Monikas?* | - Cooperation - complexitity of the structor - coomunication ways - assessment of the Intervention | | - *Is the MoniKa-Intervention used actively?* - *In summary, would you say that with Mambo and/or Monika you experience a relief in the care of multimorbid patients?* | |  |
|  | *Are there any technical difficulties during the implementation?* | - complexity of the networkfile | | - *does the network file use help in practice?* | |  |
| **6. Perspective of Mambo (5 min)** | | | | | | |
| **Transfer to the standard care** | *Are there structures that would need to be more addressed by the MamBo care model in the future?* | - Necessary changes for the transmission | - *Does Mambo emphasize an area/or areas too much so far?* - *How would you complete the following sentence: Would you recommend Mambo for regular care?* | | | - Assessment of the transmission potential |
| **communication channels** | *Would you recommend participation in Mambo to a colleague?* | - In which context - Why not? |  | | | - Do doctors advertise MamBo to their colleagues? |
| **7. Closing words and thanks** | | | | | | |
| Would you like to say or add something that has not yet been mentioned? | | | | | | - Possibility to add - Closing and adoption |
| I would like to thank all participants for coming, I look forward to the further cooperation and wish a good journey home. | | | | | |  |

## Interview-guideline focus group 2

| **Topic** | **Procedure** | | | | **Notes/ Intention** | |
| --- | --- | --- | --- | --- | --- | --- |
| **1. welcoming (5min)** | Thank you for participating in MamBo and having the time to join the focus group.  As you all know, MamBo aims to improve the outpatient care of multimorbid patients. Whether, how and why MamBo could succeed or not remains to be clarified. For this reason we have invited you today. | | | | - Entry - Introduction Mambo | |
| **2. framework (5min)** | The focus group will take around 90min. At the end there will be a catering. I will record the discussion on tape as announced so that we can evaluate your statements scientifically afterwards and do not have to rely on our memory. At the end I would like to ask you to fill out a short questionnaire about yourself, this is about socio-demographic data and how long you have been participating in Mambo.  All personal data in the interviews will be anonymized, so that no conclusions about your person or your patients will be possible. My two colleagues here will note down the change of speaker during the discussion, which will make it easier for us to transcribe later.  Do you have any further questions? | | | | - Explanation of the frame - handing out short questionnaires - Presentation of the moderator and protocolants - Consent for recording | |
| **topic** | **Main Question** | **Check** | | **Concrete request** | **Notes/ Intention** | |
| **3. Introducing**  **(5 min)** | I don't know how well you already know each other. Therefore I would like to ask you to introduce yourself briefly; your name, in which discipline you work and how long you have been participating in Mambo? | - How long in the outpatient service - How long in the Mambo Project | |  | - Get to know each other | |
| **4. Warm-Up**  **10-15 min** | We are gathered to talk about Mambo. We will also go into the individual points in more detail, but for now please tell us how is the project going; what experiences have you made so far? | - first experiences with Mambo - Perspective of general practitioners/ specialist | | - *As a medical specialist, how do you experience Mambo?* | - Do not interrupt if possible, notes for later queries - Gain first impressions of satisfaction and experiences - emotional introduction and framing | |
| 1. **Implementation of the Mambo care model (approx. 10 min)** | | | | | | |
| **Innovation-Decision-Process** | *Why did you decide to participate in MamBo?* | - Reasons for the participation - Reasons for inhibitions | | - *The physicians network has approached you actively for your participation in MamBo: How did you feel about that?* | | - ask for individual "innovation-decision-process": - knowledge, conviction/ values, decision |
| **Implementation/ Project Preparation** | *You have decided to participate in Mambo: How did it go on from there?* | - Steps of implementation - Resources | | - *What difficulties did you face before you could start?* - *What was supportive?* | | - preparation and implementation |
| 1. **evaluation of the mambo structures (ca. 30 min)** | | | | | | |
| **Patient enrollment** | *Now we want to take a closer look at the structures of Mambo.*  *How does the enrollment of the patients go?* | - Target group - particular indications - Expenses for enrollment | | - *What additional enrollment criteria for potential mambo patients could you imagine?* - *What criteria would you have in mind?* | | - Target group with the greatest benefit from a physician's perspective - Obstacles to enrollment |
| **MoniKa** | *How have you experienced the cooperation with the MoniKas so far?* | - Change in everyday practice - Changes for/with the patient - Expectations for the MoniKa-intervention | | - *How do you think patients experience the MoniKa?* | | - Complexity of use |
| **Cooperation with the MamBo-actors** | *How is the cooperation between you and the other actors of Mambo?* | - Contact regularity - physicians network, insurance - Specialists (network file) - Contact with social services | | - *How do you assess the cooperation with the care and demand management* | | - Role of the insurance (initiator/ influencer/ occupant) - Contact persons and responsibilities CM and DM helpful and useful? |
| **Continuous improvement process (CIP)** | *Can you think of any other structures that you or your practice staff have taken advantage of in the context of Mambo?* | - Awareness of the workshops - Participation yes / no why? | | - *Did the offer of workshops on drugmanagement appeal to your or your pracitce stuff?* | | - Awareness of the CIP contents - Evaluation of previous contents |
| **7. changes in everyday practice - reflection/ results (15 min)** | | | | | | |
| **Change in everyday practice life?** | *What has changed in your daily practice due to the participation in Mambo?* | - Relief - Additional effort - For example in relation to: enrollment, Monika, drug management, network file, documentation, cooperation RGL | | - *What change can you see in relation to your patients?*   *(better informed / satisfied / better cared for or greater adherence)* | | - Changes in everyday practice - Possibly only in summary the "biggest" changes - Changes in the care and health competence of patients - Confirmation, own relative advantage - Patient satisfaction |
| **8. Perspective of Mambo (5 min)** | | | | | | |
| **Transfer to the standard care** | *What is the potential of Mambo for its transfer to standard care?* | - Necessary changes for the transmission |  | | | - Assessment of the transmission potential |
| **communication channels** | *Do you talk with your colleagues about the care model MamBo?* | - In which context - Why not? | - *Would you recommend participation in Mambo to a colleague?* - *How and by whom could your colleagues be convinced most likely?* | | | - Do doctors advertise MamBo to their colleagues? - ("opinion leaders"; early adopters influence the innovation decision of late adopters (according to Rogers), |
| **9. Closing words and thanks** | | | | | | |
| Would you like to say or add something that has not yet been mentioned? | | | | | | - Possibility to add - Closing and adoption |
| I would like to thank all participants for coming, I look forward to the further cooperation and wish a good journey home. | | | | | |  |
