# Supplementary material for: When do physicians perceive the success of a new care model differently?
Source: BMC Health Serv Res. 2021 Oct 6;21:1058. doi: 10.1186/s12913-021-07061-4 (PMC8495962; doi:10.1186/s12913-021-07061-4)
Supplement: Supplementary file 2 — Final codebook. The additional file 2 indcludes a detailed description of the main categories, subcategories and their definitions. [file 12913_2021_7061_MOESM2_ESM.docx]

**When do physicians perceive the success of a new care model differently?**

A qualitative study on personal values of physicians and their expected advantages through the implementation of a new care model in a primary care setting

Authors: Simone Richter^1^, Ibrahim Demirer^1^, Maya Nocon^1^, Holger Pfaff^1^, Ute Karbach^2^

1) Institute of Medical Sociology, Health Services Research, and Rehabilitation Science (IMVR), Faculty of Human Sciences and Faculty of Medicine, University of Cologne, Germany

2) TU Dortmund University, Faculty of Rehabilitation Sciences, Germany

**Authors information**

Ibrahim Demirer: [Ibrahim.Demirer@uk-koeln.de](mailto:Ibrahim.Demirer@uk-koeln.de)

Maya Nocon: [Maya.Nocon@uk-koeln.de](mailto:Maya.Nocon@uk-koeln.de)

Holger Pfaff: Holger.Pfaff@uk-koeln.de

Ute Karbach: ute.karbach@tu-dortmund.de

**Corresponding Author**

Simone Richter

Eupener Strasse 129

50933 Cologne

Germany

Tel.+49(0)221/478-97158

Fax +49(0)221/478-1441464

Simone.Richter@uk-koeln.de

## Additional file 2

## final codebook

| Top Category | Subcategories | Definition |
| --- | --- | --- |
| Compatibility | Relevance of monetary incentives | Monetary incentives are primarily business-oriented incentives. Such as the optimisation of processes in terms of efficiency/cost-benefit ratios. |
|  | Relevance patient care/satisfaction | Participation is primarily oriented towards patient-oriented outcomes, such as the improvement of medical care, complete care, satisfaction, welfare. |
|  | Relevance of social responsibility | This refers to the subjectively perceived social responsibility towards society. Here: Doctors network, Family, Peer |
|  | Relevance of previous project experience | Previous project experiences can have a motivating but demotivating effect on the acceptance of Mambo |
| Intention to  participate | MamBo as a solution for patient-related challenges | Challenges in the care of multimorbid patients from different perspectives: 1. Challenges related to the characteristics of a multimorbid patient (impaired cognitive performance, mobility, communication, social support) 2. Aspects of the health care system that make it difficult to care for multimorbid patients and their characteristics. |
|  | MamBo as relief for non-medical tasks | Due to the physical, psychological and social conditions of multimorbid patients, additional non-medical and time-consuming tasks arise (e.g. management tasks, care of relatives, overprovision, communication problems) |
|  | MamBo as a solution for social challenges | High quality medical care in line with social requirements (family friendliness, emancipation, demography) |
|  | Mambo as a solution to avoidable costs due to incorrect and oversupply | Costs due to incorrect and over-supply can arise due to the characteristics of multimorbid patients (e.g. use of the visit to the doctor as a social contact) on the one hand, and due to defective structures in the health care system on the other (no short-term appointments, complexity of the system) |
|  | Reducing the risk of double prescription | Structural challenges in the segmented system with regard to drug safety / drug supply |
| (not) Perceived advantages of the innovation | Social management/ holistic care | Social management means, e.g. support in applying for the nursing level/ care allowance, living wills, organisation of domestic care etc. Providing a cross-sectoral holistic care, according to patients needs |
|  | Drug management | Impact on the drug management of patients |
|  | Ensuring continuous supply | Due to lack of time or holidays, for example, no home visit or adequate care can take place. Due to low mobility and autonomy of the patients, no doctor's visit can take place either. By delegating home visits in such situations, continuous care can be guaranteed. |
|  | Satisfaction (patient, doctor, relatives) | The quality of the results is connected with emotions and the satisfying commitment of MoniKa. This can refer to the practice, the staff, the patients or even the relatives. |
|  | Social health of patients | Monika's influence on the patients' social health (sociability, socialisation and participation in society) |
|  | Feeling of safety (patients at home and medical care) | A feeling of trust and security that patient care is provided at home |
|  | Stronger networking | Cooperation with various social institutions, associations (ect.) and health care institutions. |
|  | Patient information | cross-sector information gathering about patients and that patients are informed |
|  | Workload reduction/ time resources | Direct work relief of the doctor or employee through MamBo/ die MoniKa |
|  | Use of resources / resource allocation | Necessary use of resources for implementing the project and impact on the allocation of resources |
|  | Cost/benefit advantage | Resources that are put into the project vs. the benefit for the medical practice |
|  | Raising awareness of attainable tasks | Developing an awareness on the part of doctors of what needs to be delegated or what should be delegated because it exceeds their own competences. |
|  | Neutrality of the MoniKas | The MoniKa has a neutral position due to its function independent of the health insurance company and the doctor. This creates an innovative benefit in the care. |
| System-related challenges for implementation and transfer | | Cross-project challenges for the implementation and transfer of MamBo and other projects. |
